# *Pseudomonas aeruginosa* Promotes *Escherichia coli* Biofilm Formation in Nutrient-Limited Medium

**DOI:** 10.1371/journal.pone.0107186

**Published:** 2014-09-08

**Authors:** Alessandro Culotti, Aaron I. Packman

**Affiliations:** Department of Civil and Environmental Engineering, McCormick School of Engineering, Northwestern University, Evanston, Illinois, United States of America; Texas A&M University, United States of America

## Abstract

Biofilms have been implicated as an important reservoir for pathogens and commensal enteric bacteria such as *Escherichia coli* in natural and engineered water systems. However, the processes that regulate the survival of *E. coli* in aquatic biofilms have not been thoroughly studied. We examined the effects of hydrodynamic shear and nutrient concentrations on *E. coli* colonization of pre-established *Pseudomonas aeruginosa* biofilms, co-inoculation of *E. coli* and *P. aeruginosa* biofilms, and *P. aeruginosa* colonization of pre-established *E. coli* biofilms. In nutritionally-limited R2A medium, *E. coli* dominated biofilms when co-inoculated with *P. aeruginosa*, and successfully colonized and overgrew pre-established *P. aeruginosa* biofilms. In more enriched media, *P. aeruginosa* formed larger clusters, but *E. coli* still extensively overgrew and colonized the interior of *P. aeruginosa* clusters. In mono-culture, *E. coli* formed sparse and discontinuous biofilms. After *P. aeruginosa* was introduced to these biofilms, *E. coli* growth increased substantially, resulting in patterns of biofilm colonization similar to those observed under other sequences of organism introduction, i.e., *E. coli* overgrew *P. aeruginosa* and colonized the interior of *P. aeruginosa* clusters. These results demonstrate that *E. coli* not only persists in aquatic biofilms under depleted nutritional conditions, but interactions with *P. aeruginosa* can greatly increase *E. coli* growth in biofilms under these experimental conditions.

## Introduction

In aquatic environments, microorganisms coexist in complex and heterogeneous biofilm communities that exhibit genotypes and phenotypes distinct from their planktonic counterparts [1]–[3]. Biofilms are of particular concern in engineered water distribution systems, as they have been found to harbour pathogens as diverse as enteric viruses, cysts of protozoa such as *Cryptosporidium parvum* and *Giardia lamblia*, and bacteria such as *Pseudomonas aeruginosa*, *Escherichia coli*, *Campylobacter jejuni*, *Helicobacter pylori*, *Legionella pneumophila,* and *Aeromonas* spp. [1], [3]–[5]. Residence within biofilms can provide microorganisms with access to higher concentrations of nutrients and protection from environmental stresses and chemical disinfectants [1], [2], [6], [7]. Biological interactions that occur between newly-associated pathogens and indigenous microbial populations have been shown to regulate pathogen persistence and growth [1], [6]–[8]. Prior studies on the survival of biofilm-associated pathogens [9]–[12] suggest that biofilms may play an especially important role in the persistence and dissemination of fastidious and stress-sensitive organisms in the environment [1]. A deeper understanding of the survival and growth potential of pathogens and fecal indicator organisms in aquatic biofilms is needed for the continued improvement of water treatment strategies and identification of potential sources of pathogen contamination.

Historically, *E. coli* has been used as an indicator of fecal contamination in the United States and elsewhere [13], [14]. For years, *E. coli* was believed to survive poorly outside of a living host and not grow in secondary habitats due to a constant exposure to environmental stresses [15]. However, recent reports have shown that *E. coli* can survive and potentially replicate in nutrient-rich aquatic environments [15]. Mounting evidence also suggests that *E. coli* and other enteric organisms can persist and potentially grow in drinking water distribution networks [16], [17]. Laboratory-scale studies have demonstrated that *E. coli* can become associated with pre-established indigenous biofilms of the type found in drinking water distribution systems [6], [18]. The prolonged survival of *E. coli* in aquatic biofilms may lead to the incorrect detection of fecal contamination and mask true breakthrough events in water distribution networks. Temperature, nutrient availability, concentration of disinfectants and antagonistic bacterial predation have all been shown to influence the persistence and growth of *E. coli* in aquatic environments [6], [7], [15], [19]. However, little is known of the conditions under which *E. coli* can colonize pre-existing aquatic biofilms, or the mechanisms of their interaction with typical aquatic biofilm-forming organisms.

Here, we investigated the effects of varying nutrient concentrations and fluid velocities on the ability of *E. coli* to form biofilms in co-culture with the robust and ubiquitous aquatic biofilm-forming bacterium *Pseudomonas aeruginosa*. To evaluate the effects of timing of organism introduction, we investigated *E. coli* colonization of pre-established *P. aeruginosa* biofilms, co-inoculation of *E. coli* and *P. aeruginosa* biofilms, and *P. aeruginosa* colonization of pre-established *E. coli* biofilms. Additionally, we examined the role of indole, an *E. coli* metabolite, in regulating *E. coli* and *P. aeruginosa* interactions in biofilms.

## Experimental Procedures

### Flow cells

Biofilm growth was observed using a 2-D planar flow cell, and a single-channel microfluidic flow cell [20]. The planar flow cell was used for the study of biofilms under a distribution of local velocities in a single experiment. The flow chamber is 35 mm×35 mm×0.6 mm in size, and consists of a transparent acrylic base and a glass coverslip. The glass coverslip allows for direct observations of biofilm development by microscopy. Controlled patterns of inflow can be imposed by means of inflow or outflow ports distributed around the periphery of the flow cell. Here, flow was introduced uniformly to the inflow ports on one side of the flow cell, producing a right-angle turning flow that imposes a distribution of velocities and medium flux rates over the biofilm. Nine regions (R1–9) at the center of the flow chamber were selected for biofilm imaging, forming a 10 mm×10 mm observation window. A detailed description of the flow cell design and performance can be found in Zhang *et al*. [20].

A polydimethylsiloxane (PDMS), single-channel microfluidic flow cell was used to study biofilm growth under a controlled, uniform velocity field. Each channel measures 35 mm×4 mm×1 mm in size, with a single inlet and outlet port. A glass coverslip at the base of each channel allows for *in* *situ* visualization of the biofilms. A detailed description of the flow cell design can be found in Song *et al.* [21].

### Bacterial strains

Biofilm flow cell experiments were performed with several *E. coli* strains and a single typical *P. aeruginosa* biofilm-forming strain. An *E. coli* DH5α strain inserted with plasmid pUC encoding mCherry fluorescent proteins was used to study dual-species biofilm growth behavior. This strain is derived from *E. coli* K-12. The indole-deficient *tnaA* single deletion mutant *E. coli* JW3686 [22] and its parent strain *E. coli* BW25113 [22] were used to study the effects of indole on dual-species biofilm growth. Both *E. coli* JW3686 and *E. coli* BW25113 are derivatives of *E. coli* K-12. A *P. aeruginosa* PAO1 strain with a chromosomally expressed green fluorescent protein (GFP) was also used. *P. aeruginosa* PAO1 is the definitive laboratory model strain used for biofilm research. Stock cultures of *P. aeruginosa* and *E. coli* were streaked onto Luria-Bertani (LB) agar plates, and incubated for 24 hours at 37°C. Single colonies of each strain were transferred into separate tubes containing 3 mL of sterile LB broth, and grown overnight in a shaker at 37°C and 225 rpm for injection into the flow cells.

### Flow cell experimental conditions

Mono- and mixed-culture biofilm experiments were run using R2A media at room temperature (24°C). R2A was chosen as a nutritionally depleted medium. R2A consists of 0.05 g/L yeast extract, 0.05 g/L proteose peptone No. 3, 0.05 g/L casamino acids, 0.05 g/L dextrose, 0.03 g/L sodium pyruvate, 0.03 g/L dipotassium phosphate, and 0.005 g/L magnesium sulfate [9]. This medium has most typically been used to grow and enumerate microorganisms from drinking water sources [23]. Gilson Miniplus 3 peristaltic pumps were used to circulate the R2A medium, as they produce minimal flow pulsations and are well suited for biofilm experiments. The flow was regulated to 0.8 mL/min (0.2 mL/min per inflow port) in planar flow cells. We chose this flow configuration because the resulting velocity gradient (0.96–1.74 mm/s) has been shown to produce distinct growth and detachment patterns in *P. aeruginosa* biofilms [20]. In the microfluidic flow cells, the flow was regulated to 0.2 mL/min. All experiments were replicated three times using independent flow cells run in parallel.

To investigate the colonization of pre-existing *P. aeruginosa* biofilms by *E. coli* in planar and microfluidic flow cells, 1 mL of a stationary-phase culture of *P. aeruginosa* (OD_600_ = 0.1) was first injected into each chamber and allowed to deposit on the flow cell coverslip under stagnant conditions for one hour. The flow of standard R2A was then initiated and maintained at a constant rate for 3 days. On day 3, the flow cells containing pre-established *P. aeruginosa* biofilms were inoculated using 1 mL of stationary-phase *E. coli* cultures (OD_600_ = 0.1), and the flow was halted for 30 min to facilitate *E. coli* deposition into the biofilm. The inflow of medium was then resumed and maintained at a constant rate for an additional 3 days. These experiments were also conducted using 4x and 8x concentrated R2A medium in microfluidic flow cells to investigate the effects of nutritional conditions on growth of biofilm co-cultures.

In co-inoculated experiments, 0.5 mL of stationary-phase cultures of *E. coli* and *P. aeruginosa* were diluted to an OD_600_ of 0.1 and mixed at a ratio of 1∶1 (1 mL total volume). The cell mixtures were then inoculated in both planar and microfluidic flow cells. Inoculation was followed by a 1-h stagnant period to facilitate attachment of cells, and the flow of standard R2A medium was then resumed for 3 days. In microfluidic flow cells, these experiments were also conducted using 4x and 8x concentrated R2A medium.

To investigate the colonization of pre-existing *E. coli* biofilms by *P. aeruginosa*, 1 mL of a stationary-phase culture of *E. coli* (OD_600_ = 0.1) was injected into a microfluidic flow cell with standard R2A medium and allowed to attach on the glass coverslip under stagnant conditions for one hour. The inflow of R2A medium was then initiated and maintained at a constant rate for a period of 3 days. On day 3, the flow chambers containing pre-established *P. aeruginosa* biofilms were inoculated using 1 mL of stationary-phase cultures of *P. aeruginosa* (OD_600_ = 0.1), and the flow was halted for 30 min to facilitate *P. aeruginosa* deposition. The flow was then resumed and maintained at a constant rate for 3 additional days.

### Batch experiments

Batch cultures were grown for comparison with biofilm results. Pure cultures of *P. aeruginosa* and *E. coli* were grown in LB medium overnight at 24°C, and subsequently diluted to an OD_600_ of 0.1 (1.6×10^8^ CFU/mL). Erlenmeyer flasks containing 20 mL of sterile R2A medium were inoculated using 150 µL of each culture. The mixed cultures were incubated in a shaker (150 rpm) at 24°C overnight. Each day for 3 days, 300 µL of the mixed culture was transferred into a new Erlenmeyer flask containing 20 mL of fresh R2A. The batch experiments were repeated using an incubation temperature of 37°C. These mixed batch cultures were sampled daily for CFU using selective media in order to differentiate *P. aeruginosa* and *E. coli*. Ampicillin (100 µg/mL) was used to select for *P. aeruginosa*, and cefsulodin (20 µg/mL) was used to select for *E. coli* [24]. Batch cultures were repeated in triplicate.

### Imaging procedures

Biofilm micrographs were obtained using a Leica SP5 confocal laser scanning microscope, and collected with Leica Confocal Software. The three-dimensional biofilm images were generated from the planar image stacks using the image processing software VOLOCITY (Improvision, Inc.) Quantitative analysis of the biofilm structures was conducted using the COMSTAT image processing software [25].

Antibiotic selection for mCherry plasmid-encoding *E. coli* was not performed in flow cell experiments to avoid negative effects on *P. aeruginosa* in mixed biofilms. As a result, mCherry fluorescence was lost after a period of 18–24 hours. Imaging of *E. coli* using mCherry fluorescence was therefore only used during the initial cell deposition and attachment phase. *E. coli* was subsequently imaged by counterstaining the mixed biofilms using SYTO 62, a cell-permeant nucleic acid stain (Life Technologies). *P. aeruginosa* was imaged by constitutively expressed gfp fluorescence. Biofilms counterstaining was conducted at the end of each experiment using a 50 µM solution of SYTO 62 for a period of 30 minutes in the dark. The flow was then resumed for 20 min in order to wash out unbound stain.

## Results

### Colonization of pre-established *P. aeruginosa* biofilms by *E. coli*

**Figure 1:**
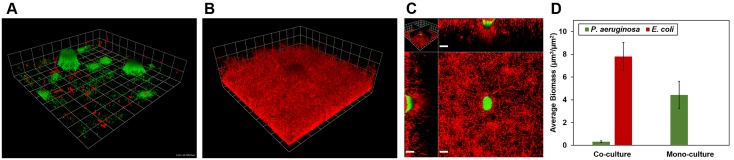
Colonization of *P. aeruginosa* biofilms by *E. coli* in R2A medium in a microfluidic flow cell. *E. coli* appears red and *P. aeruginosa* appears green or yellow. **A)** *E. coli* deposited on 3-day old *P. aeruginosa* biofilm (grid unit is 23.8 µm). **B)** Resulting mixed *E. coli* – *P. aeruginosa* biofilm on day 6 (grid unit is 23.8 µm). **C)** Horizontal section near the base of the biofilm and vertical sections of the biofilm shown in panel B (scale bar = 20 µM). **D)** *P. aeruginosa* and *E. coli* biomass in mixed biofilms on day 6 compared with biomass of 6-day old mono-cultured *P. aeruginosa* biofilms.

*E. coli* colonization of pre-existing, 3-day-old *P. aeruginosa* biofilms is shown in Fig. 1. *P. aeruginosa* biofilms grew in clusters with scattered cells present between colonies (Fig. 1A). Within three days of introduction, *E. coli* was able to extensively colonize the biofilm (Fig. 1B, C). *E. coli* overgrew *P. aeruginosa* clusters and colonized the interior of clusters as well (Fig. 1C). The average *E. coli* biomass was consistently greater than that of *P. aeruginosa* (Fig. 1D). Moreover, we found substantially less *P. aeruginosa* in mixed culture compared to mono-species biofilms on day 6 (Fig. 1D). This indicates that *E. coli* not only dominated in mixed culture, but also hindered *P. aeruginosa* growth. These results show that *E. coli* can successfully colonize, outcompete, and outgrow established *P. aeruginosa* biofilms in the nutritionally limited R2A medium.

### Co-inoculation of *P. aeruginosa* and *E. coli*

**Figure 2:**
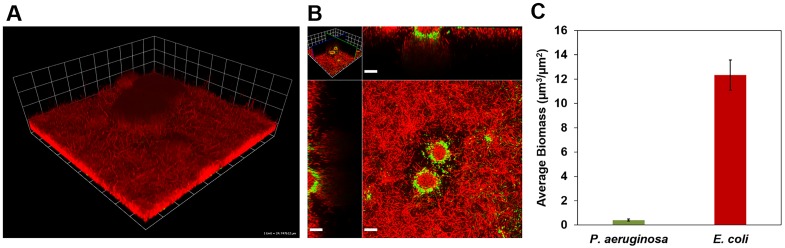
Co-development of *E. coli* and *P. aeruginosa* biofilms in a microfluidic flow cell and R2A medium. *E. coli* appears red and *P. aeruginosa* appears green or yellow. **A)** 3-day old co-inoculated *P. aeruginosa* - *E. coli* biofilm (grid unit is 23.8 µm). **B)** Horizontal section near the base of the biofilm and vertical sections of the biofilm shown in panel A (scale bar = 20 µM). **C)** *P. aeruginosa* and *E. coli* biofilm biomass on day 3.

Growth of co-inoculated, 3-day-old biofilms in microfluidic flow cells is shown in Fig. 2. *E. coli* grew extensively in a lawn-like fashion (Fig. 2A), and consistently over-grew *P. aeruginosa*, which was present primarily in isolated clusters (Fig. 2B). *E. coli* also successfully colonized the interior of the *P. aeruginosa* clusters, restricting *P. aeruginosa* to a thin shell near the exterior of each cluster. The average biomass data presented in Fig. 2C highlights the overwhelming success of *E. coli* in colonizing the landscape compared to *P. aeruginosa*. Conversely, *P. aeruginosa* consistently achieved greater population density than *E. coli* in batch co-cultures (Fig. S1, S2 in File S1). These differences emphasize that population growth dynamics and competition for resources change substantially in biofilms.

### Effects of nutrient concentrations on the colonization of pre-established *P. aeruginosa* biofilms by *E. coli*

**Figure 3:**
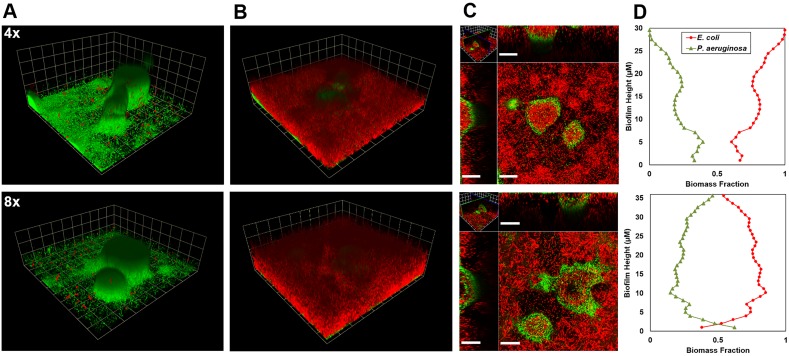
Colonization of *P. aeruginosa* biofilms by *E. coli* under varying R2A medium concentrations in a microfluidic flow cell. *E. coli* appears red and *P. aeruginosa* appears green. Column A) *E. coli* deposited on 3-day old mono-species *P. aeruginosa* biofilms under 4x and 8x R2A concentrations (grid unit is 23.8 µm). B) *P. aerugiosa* and *E. coli* biofilms on day 6, 3 days after introduction of *E. coli* (grid unit is 23.8 µm). C) Horizontal section near the base of the biofilm and vertical sections of the biofilm shown in panel B (scale bar = 40 µm). D) Biomass fraction vs. biofilm height graph. *P. aeruginosa* biofilm biomass increased with R2A concentration. *E. coli* dominated the community under all medium concentrations regardless of the *P. aeruginosa* cluster morphology. Biofilms were counter-stained by SYTO 62.

**Figure 4:**
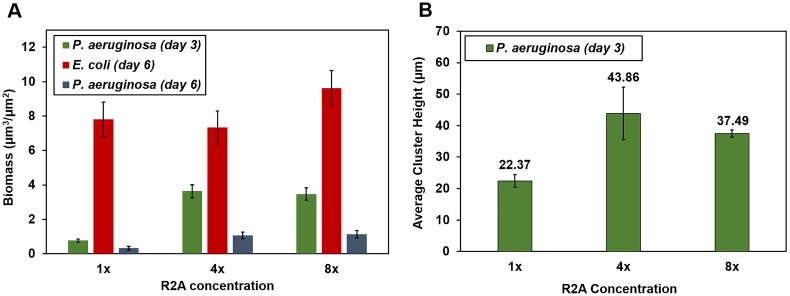
*E. coli* colonization of pre-established *P. aeruginosa* biofilms under varying R2A medium concentrations. **A)** Average biofilm biomass vs. R2A concentration for 1) *P. aeruginosa* on day 3, 2) *E. coli* on day 6, and 3) *P. aeruginosa* on day 6. **B)** Average *P. aeruginosa* cluster height vs. R2A concentration on day 3.

Larger and morphologically distinct *P. aeruginosa* biofilms grew in enriched R2A medium (Fig. 3A). Significantly more *P. aeruginosa* biomass and taller clusters grew under enriched 4x and 8x R2A media relative to the standard (depleted) R2A medium (t-test P<0.01) (Fig. 4). However, differences in biomass between 4x and 8x R2A media were not significant (P>0.60). *P. aeruginosa* biomass decreased after *E. coli* inoculation. *P. aeruginosa* biomass in mixed culture on day 6 (3 days after *E. coli* inoculation) was consistently lower than on day 3 (at the time of *E. coli* inoculation) for all nutritional conditions.

*E. coli* grew to substantially greater biomass than *P. aeruginosa* within 3 days of inoculation (Fig. 4B). Unexpectedly, *E. coli* biomass was not significantly different under enriched conditions (4x and 8x R2A) compared to standard R2A (P>0.50 and P>0.09 respectively). The biofilm morphology of *E. coli* did not vary, with lawn-like growth throughout the chamber and colonization of the interior of *P. aeruginosa* clusters under all conditions tested (Fig. 3B, C). *P. aeruginosa* clusters were readily colonized by *E. coli*, with substantial intergrowth occurring even in the largest and tallest *P. aeruginosa* clusters (Fig. 3C). Despite greater *P. aeruginosa* growth under more nutritionally rich conditions, mixed biofilms were still dominated by *E. coli* throughout the entire biofilm height (Fig. 3D). Similar patterns of colonization were observed in co-cultured biofilms as well (Fig. S3, S4 in File S1).

### Effects of varying fluid velocities on colonization of pre-established *P. aeruginosa* biofilms by *E. coli*

**Figure 5:**
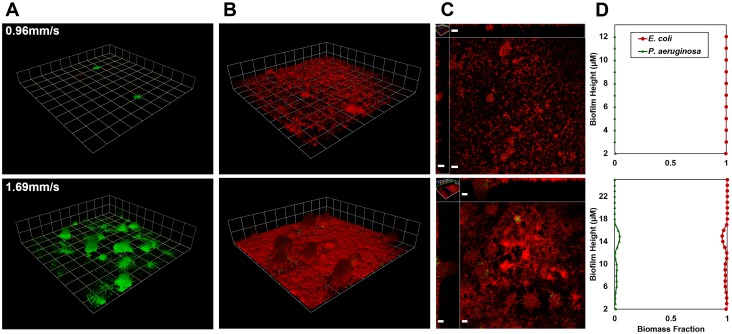
Colonization of *P. aeruginosa* biofilms by *E. coli* under a controlled flow gradient in a planar flow cell and in R2A medium. *E. coli* appears red and *P. aeruginosa* appears green. Results are presented for two local fluid velocities, 0.96 mm/s (top row) and 1.69 mm/s (bottom row). Column **A)** *E. coli* deposited on 3-day old *P. aeruginosa* biofilms (grid unit is 23.8 µm). **B)** Mixed *P. aerugiosa* and *E. coli* biofilms on day 6, 3 days after *E. coli* inoculation (grid unit in B is 23.8 µm). **C)** Horizontal section near the base of the biofilm and vertical sections of the biofilm shown in panel B (scale bar = 15 µm). **D)** Distribution of *P. aerugiosa* and *E. coli* biomass as function of height, indicating that *E. coli* was the dominant species throughout the biofilm. *E. coli* appears red and *P. aeruginosa*-GFP appears green.

**Figure 6:**
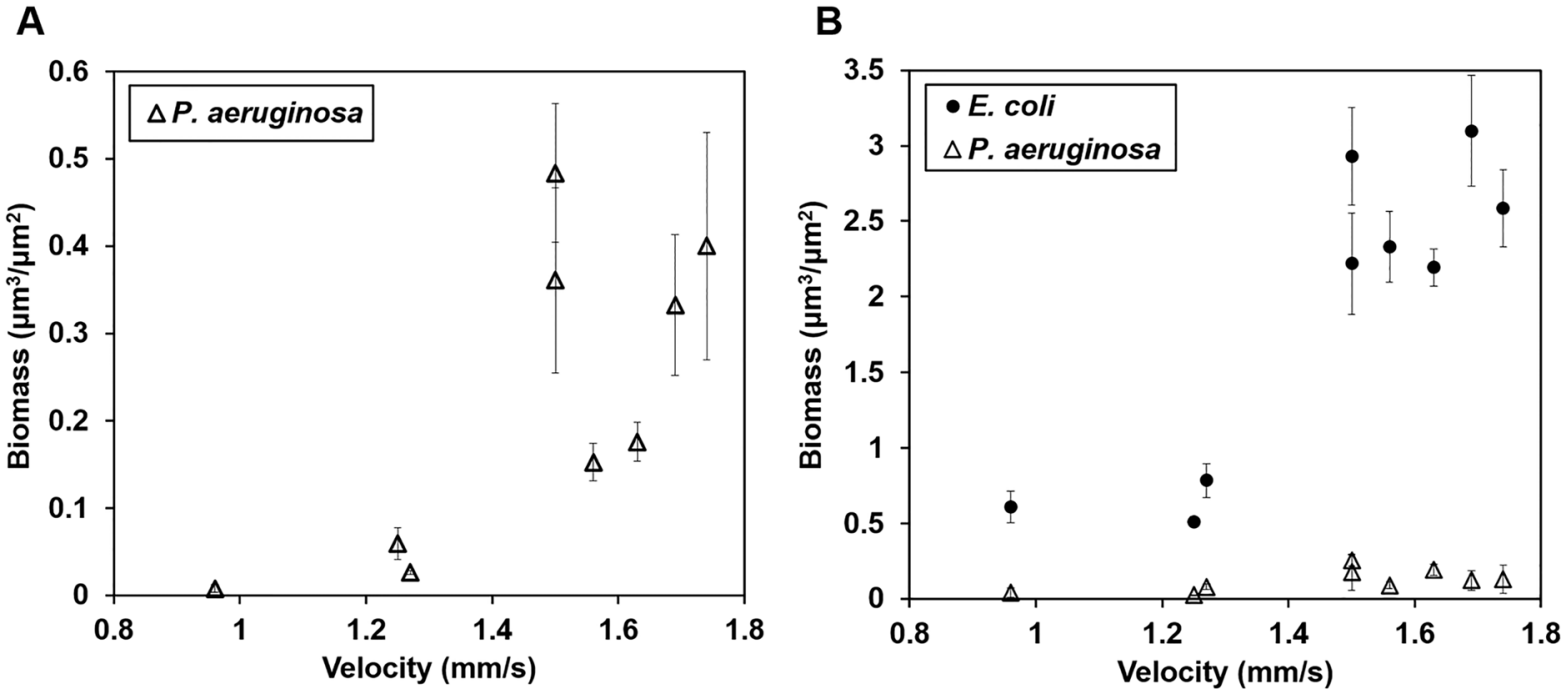
Biomass of *P. aeruginosa* and *E. coli* biofilms subjected to different local velocities in R2A medium. **A)** Average biomass vs. fluid velocity for mono-cultured, 3-day old *P. aeruginosa* biofilms. **B)** Average biomass vs. fluid velocity on day 6 of experiments on colonization of *P. aeruginosa* by *E. coli* (after 3 days of mono-culture *P. aeruginosa* growth plus an additional 3 days after introduction of *E. coli*).

Higher local fluid velocities increased the growth of both *P. aeruginosa* and *E. coli* in biofilms (Fig. 5, 6). *P. aeruginosa* grew to significantly greater biomass in regions of higher velocity (1.50–1.74 mm/s) than in regions of lower velocity (0.96–1.27 mm/s), both on day 3 (P<0.001) and day 6 (P<0.01). *E. coli* strongly dominated biofilm biomass on day 6, 3 days after its inoculation, in all regions of the flow cell regardless of local fluid velocities (Fig. 5B, 6B). *E. coli* grew consistently in a lawn-like fashion, overgrowing *P. aeruginosa* clusters and also colonizing the interior of clusters. *E. coli* was the dominant species over the full height of the biofilm (Fig. 5D). *E. coli* also developed greater biomass in regions of higher velocity (P<0.001). Similar trends with velocity were also observed in co-inoculation experiments (Fig. S5, S6 in File S1).

### Colonization of *E. coli* biofilms by *P. aeruginosa*

**Figure 7:**
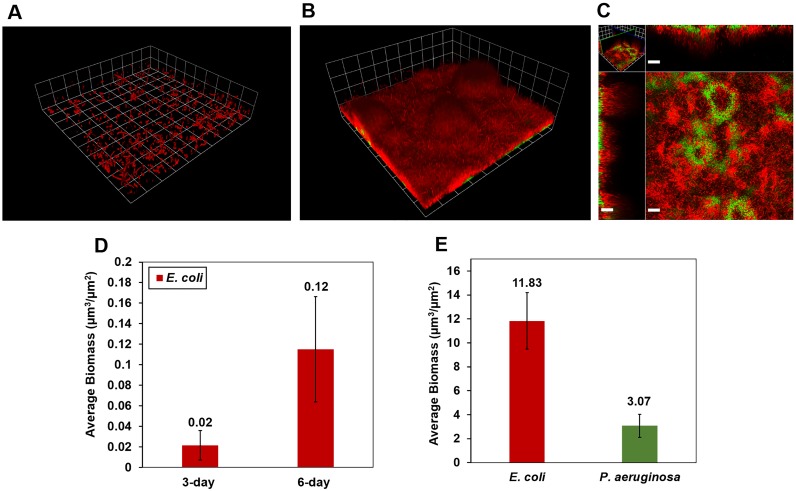
Colonization of *E. coli* biofilms by *P. aeruginosa* in R2A medium. *E. coli* appears red and *P. aeruginosa*-GFP appears green or yellow. **A)** After 3-days of mono-species growth, *E. coli* formed sparse biofilms composed of small, isolated cell clusters (grid unit is 23.8 µm). **B)** Mixed *P. aeruginosa* and *E. coli* biofilm on day 6, after 3 days of mono-species *E. coli* growth plus 3 additional days of multi-species growth after inoculation of *P. aeruginosa*. (grid unit in B is 23.8 µm). Following introduction of *P. aeruginosa*, *E. coli* grew prolifically and adopted a configuration similar to that observed in other mixed-species experiments. **C)** Horizontal section near the base of the biofilm and vertical sections of the biofilm shown in panel B (scale bar = 20 µm). **D)** Biomass of 3-day- and 6-day-old *E. coli* mono-cultured biofilms. **E)** Biomass of co-developed biofilms after 3 days of *E. coli* growth plus 3 additional days of multi-species growth after inoculation of *P. aeruginosa*.

The *E. coli* strain used in this study formed biofilms poorly in mono-culture. After 3 days of growth, *E. coli* only formed sparse, discontinuous biofilms (Fig. 7A, D). Similarly limited *E. coli* growth occurred after 6 days of monospecies culturing (Fig. 7D). However, the introduction of *P. aeruginosa* caused a dramatic increase in *E. coli* growth (Fig. 7B, C). On day 6 of the colonization experiments, 3 days after the inoculation of *P. aeruginosa* on 3-day old *E. coli*, *P. aeruginosa* grew into discrete clusters, and *E. coli* again grew into an extensive lawn and colonized the interior and exterior of *P. aeruginosa* clusters (Fig. 7C). As a result, *E. coli* biofilm biomass after 3 days of mono-culture growth plus 3 days after introduction of *P. aeruginosa* was substantially greater than the *E. coli* biofilm biomass after 6 days of mono-culture growth (P<0.001) (Fig. 7E, F). These findings demonstrate that the introduction of *P. aeruginosa* can enhance the growth of *E. coli* and facilitate the formation of biofilms.

### Colonization of indole-deficient *E. coli* biofilms by *P. aeruginosa*

**Figure 8:**
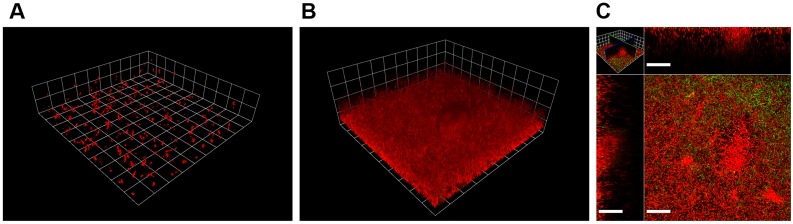
Colonization of indole-deficient *E. coli* JW3686 biofilms by *P. aeruginosa* in R2A medium. *E. coli* appears red and *P. aeruginosa*-GFP appears green or yellow. **A)** Mono-cultured, 3-day old *E. coli* formed sparse biofilms composed of small, isolated cell clusters (grid unit is 23.8 µm). **B)** Mixed *P. aeruginosa* and *E. coli* biofilm on day 6, after 3 days of mono-species *E. coli* growth plus 3 additional days of multi-species growth after inoculation of *P. aeruginosa*. (grid unit in B is 23.8 µm). Following introduction of *P. aeruginosa*, *E. coli* grew extensively and adopted a configuration similar to that observed in other mixed-species experiments. **C)** Horizontal section near the base of the biofilm and vertical sections of the biofilm shown in panel B (scale bar = 40 µm).

**Figure 9:**
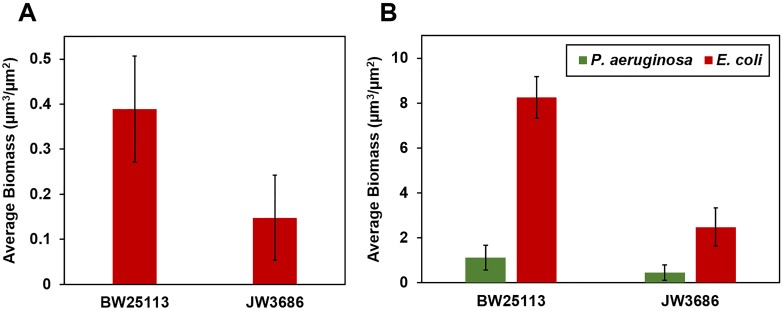
Biomass of *E. coli* JW3686 and BW25113 biofilms in mono-culture and after colonization by *P. aeruginosa* in R2A medium. **A)** Average biomass of mono-cultured *E. coli* on day 3. **B)** Average biomass of *E. coli* and *P. aeruginosa* in mixed biofilms on day 6, 3 days after introduction of *P. aeruginosa*.

Mono-cultured *E. coli* JW3686 formed sparse and discontinuous biofilms over a 3-day growth period (Fig. 8A, 9A). After the introduction of *P. aeruginosa* however, *E. coli* grew rapidly in the mixed-species biofilms. By day 6 of the experiment, 3 days after *P. aeruginosa* inoculation, *E. coli* formed lawn-like biofilms with isolated larger colonies interspersed with *P. aeruginosa* (Fig. 8B, C, 9B). Control experiments using the indole-positive parent strain, *E. coli* BW25113, also resulted in very similar mono- and dual-species biofilm growth (Fig. 9A, B, and Fig. S7 in File S1). Mono-cultured *E. coli* BW25113 formed biofilms poorly over a 3-day growth period (Fig. S7A in File S1), but the introduction of *P. aeruginosa* triggered a rapid growth response that enabled *E. coli* to outcompete and colonize the *P. aeruginosa* biofilms (Fig. 9B, and Fig. S7B, C in File S1).

## Discussion

We investigated the effects of hydrodynamic shear and nutrient concentrations on *E. coli* colonization of pre-established *Pseudomonas aeruginosa* biofilms, co-inoculation of *E. coli* and *P. aeruginosa* biofilms, and *P. aeruginosa* colonization of pre-established *E. coli* biofilms. In R2A medium, *P. aeruginosa* formed robust biofilms with continuous surface coverage and mound-shaped clusters, as is typical of this organism under a wide range of environmental conditions [26], [27]. Conversely, *E. coli* only formed sparse biofilms with very small, discontinuous microcolonies in mono-culture. Many *E. coli* strains are known to be poor biofilm formers [28], [29]. In particular, *E. coli* K-12 strains lacking the F episome, a plasmid responsible for conjugative pili [26], and strains with low cell motility exhibit poor biofilm growth [28], [30], [31]. Because the *E. coli* DH5α strain used in this study does not carry the F episome and is not highly motile [28], [32], we expected minimal *E. coli* biofilm growth. However, the introduction of *P. aeruginosa* triggered a growth response that enabled extensive lawn-like biofilm formation by *E. coli*.

Deposited *E. coli* consistently overgrew pre-established *P. aeruginosa* biofilms and also colonized the interior of *P. aeruginosa* clusters under nutrient-limited conditions. We also observed this behavior in co-inoculated experiments, and in experiments where *P. aeruginosa* was introduced to pre-established *E. coli* biofilms. The colonization of the cluster interiors by *E. coli* is likely associated with the process of coordinated cell dispersal in *P. aeruginosa* biofilms. During these dispersal events, cells actively evacuate the interior of *P. aeruginosa* clusters through breaches in the cluster wall [33] and leave behind hollow, shell-like structures [33]–[36]. The “hollowing” of bacterial microcolonies from coordinated cell evacuation has been previously documented, though the suspected mechanisms responsible for these events differ from species to species [33], [36]–[39]. The transfer of exogenous solutes into the interior regions of the biofilm clusters has also been suggested to regulate dispersal events [33]. Because we observed void formation in *P. aeruginosa* biofilms under all experimental conditions tested, this phenomenon appears to generally facilitate *E. coli* colonization by providing access to the interior of biofilm cell clusters.

*P. aeruginosa* grew substantially less in mixed culture than in mono-culture under identical nutritional and flow conditions. Moreover, *P. aeruginosa* biofilm biomass decreased following introduction of *E. coli*. The reduction of *P. aeruginosa* biomass in dual-species biofilms suggests strong antagonistic behavior by *E. coli* towards *P. aeruginosa*. These results were surprising, as *P. aeruginosa* is known to be a robust biofilm-forming microorganism in a wide range of aquatic environments, and can produce a variety of antimicrobial agents that adversely affect the growth of other organisms in biofilms [40]–[42]. However, similar overgrowth of *P. aeruginosa* in dual-species biofilms with *Flavobacterium* has also been reported by Zhang et al. [43]. By comparison, *P. aeruginosa* consistently outgrew *E. coli* in batch experiments, demonstrating that biofilm growth can modify inter-species competition and the relative growth rates of individual species in mixed culture.

Accumulation of extracellular indole has been previously reported to decrease *E. coli* biofilm formation by hindering cell motility [45], [47]. Indole is an *E. coli* metabolite that is produced from the amino acid tryptophan by the enzyme tryptophanase [46]. *P. aeruginosa* degrades indole [24], and can potentially enhance *E. coli* biofilms by eliminating the growth inhibition caused by extracellular indole [45], [48]. However, under the conditions imposed in this study, we found that indole-producing and indole-deficient strains of *E. coli* grew similarly in biofilms. The indole-deficient *E. coli* mutant JW3686 (*tnaA*) grew poorly in mono-culture, but was able to form extensive lawn-like biofilms after the introduction of *P. aeruginosa*. We observed the same growth behavior in experiments using the indole-producing strains *E. coli* BW25113 and *E. coli* DH5α. The most prominent effects of indole on *E. coli* and *P. aeruginosa* have been observed at physiologically relevant concentrations between 500 µM and 1 mM, in media that is rich with exogenous tryptophan; e.g., LB medium [24], [44], [45], [47]. However, the tryptophan content in R2A medium (2.3–2.5 µM) is substantially less than that of LB medium (510 µM) (BD Bionutrients Technical Manual Third Edition). This suggests that indole may play a larger role in the growth behavior of *E. coli* – *P. aeruginosa* biofilms only in media that contains greater concentrations of tryptophan. We also show that the triggered *E. coli* biofilm growth phenomenon after introduction of *P. aeruginosa* is not limited to *E. coli* DH5α, but can also occur in other *E. coli* strains including BW25113 and the indole-deficient JW3686 mutant.

Biofilm growth was more extensive under faster local velocities and in more nutrient-rich R2A media (4x and 8x concentrations). In regions of faster flow, increased biofilm growth occurred for both *P. aeruginosa* and *E. coli*. This behavior has been previously attributed to more rapid delivery of substrates and nutrients to biofilms under higher local velocities [20]. In more concentrated R2A media, increased growth occurred primarily in *P. aeruginosa* biofilms, but not *E. coli*. This suggests that, unlike *P. aeruginosa*, the growth of *E. coli* was not constrained by nutrient availability in the R2A medium. However, *E. coli* remained the dominant species in co-culture under all velocities and media tested regardless of *P. aeruginosa* biofilm growth and morphology. *E. coli* consistently colonized outer regions of *P. aeruginosa* clusters and extensively colonized the larger and taller clusters. The results demonstrate that *E. coli* can outcompete *P. aeruginosa* in dual-species biofilms under nutrient-depleted conditions.

In conclusion, we demonstrated that *E. coli* is capable of colonizing and overgrowing *P. aeruginosa* biofilms under both nutrient-limited and enriched conditions, and over a range of fluid velocities. We further showed that the introduction of *P. aeruginosa* triggered a dramatic growth response in *E. coli* that enabled extensive biofilm formation. By comparison, *P. aeruginosa* outgrew *E. coli* in co-cultured planktonic batch experiments, illustrating that biofilm growth substantially modifies inter-species competition and growth rates. These results show that interactions with pre-established bacterial biofilms such as *P. aeruginosa* can greatly increase the ability of *E. coli* to persist and grow in aquatic environments.

## Supporting Information

File S1 **Figures S1–S7.** Figure S1. Mean population density of *E. coli* and *P. aeruginosa* grown in R2A medium over a 3-day period at 37°C. Both populations remained steady for the duration of the experiment, with *P. aeruginosa* exhibiting a higher population concentration. Figure S2. Mean population density of *E. coli* and *P. aeruginosa* grown in R2A medium over a 3-day period at 24°C. Both populations remained relatively steady for the duration of the experiment, with *P. aeruginosa* exhibiting a higher population concentration. Figure S3. Co-development of 3-day old *E. coli* and *P. aeruginosa* biofilms under varying R2A medium concentrations in a microfluidic flow cell. Column: A) 3-day old co-inoculated *P. aeruginosa* – *E. coli* biofilms with under 4x and 8x R2A concentrations (grid unit is 23.8 µm). B) Horizontal section near the base of the biofilm and vertical sections of the biofilm shown in panel A (scale bar = 40 µm). C) Biomass fraction vs. biofilm height graph. Biofilms were counter-stained by SYTO 62. *E. coli* appears red and *P. aeruginosa*-GFP appears green. Figure S4. Average biofilm biomass vs. R2A concentration for co-development of 3-day old *E. coli* and *P. aeruginosa* biofilms. Figure S5. Co-development of 3-day old *E. coli* and *P. aeruginosa* biofilms under a controlled flow gradient in a planar flow cell and in R2A medium. Results are presented for two local fluid velocities, 0.96 mm/s (top row) and 1.69 mm/s (bottom row). Column: A) 3-day old co-inoculated *P. aeruginosa* – *E. coli* biofilms (grid unit is 23.8 µm). B) Horizontal section near the base of the biofilm and vertical sections of the biofilm shown in panel A (scale bar = 20 µm). C) Distribution of *P. aerugiosa* and *E. coli* biomass as function of height, indicating that *E. coli* was the dominant species throughout a majority of the biofilm. *E. coli* appears red and *P. aeruginosa*-GFP appears green. Figure S6. Biofilm biomass on day 3 for the co-development of *P. aeruginosa* and *E. coli* biofilms subjected to different local velocities in R2A medium. Figure S7. Colonization of *E. coli* BW25113 biofilms by *P. aeruginosa* in R2A medium. *E. coli* appears red and *P. aeruginosa*-GFP appears green or yellow. A) After 3-days of mono-species growth, *E. coli* formed sparse biofilms composed of small, isolated cell clusters (grid unit is 23.8 µm). B) Mixed *P. aeruginosa* and *E. coli* biofilm on day 6, after 3 days of mono-species *E. coli* growth plus 3 additional days of multi-species growth after inoculation of *P. aeruginosa*. (grid unit in B is 23.8 µm). Following introduction of *P. aeruginosa*, *E. coli* grew prolifically and adopted a configuration similar to that observed in other mixed-species experiments. C) Horizontal section near the base of the biofilm and vertical sections of the biofilm shown in panel B (scale bar = 40 µm).
(DOCX)
