# Supplementary material for: Pseudomonas aeruginosa Promotes Escherichia coli Biofilm Formation in Nutrient-Limited Medium
Source: PLoS One. 2014 Sep 8;9(9):e107186. doi: 10.1371/journal.pone.0107186 (PMC4157881; doi:10.1371/journal.pone.0107186)
Supplement: File S1 — Figures S1–S7. Figure S1. Mean population density of E. coli and P. aeruginosa grown in R2A medium over a 3-day period at 37°C. Both populations remained steady for the duration of the experiment, with P. aeruginosa exhibiting a higher population concentration. Figure S2. Mean population density of E. coli and P. aeruginosa grown in R2A medium over a 3-day period at 24°C. Both populations remained relatively steady for the duration of the experiment, with P. aeruginosa exhibiting a higher population concentration. Figure S3. Co-development of 3-day old E. coli and P. aeruginosa biofilms under varying R2A medium concentrations in a microfluidic flow cell. Column: A) 3-day old co-inoculated P. aeruginosa – E. coli biofilms with under 4x and 8x R2A concentrations (grid unit is 23.8 µm). B) Horizontal section near the base of the biofilm and vertical sections of the biofilm shown in panel A (scale bar = 40 µm). C) Biomass fraction vs. biofilm height graph. Biofilms were counter-stained by SYTO 62. E. coli appears red and P. aeruginosa-GFP appears green. Figure S4. Average biofilm biomass vs. R2A concentration for co-development of 3-day old E. coli and P. aeruginosa biofilms. Figure S5. Co-development of 3-day old E. coli and P. aeruginosa biofilms under a controlled flow gradient in a planar flow cell and in R2A medium. Results are presented for two local fluid velocities, 0.96 mm/s (top row) and 1.69 mm/s (bottom row). Column: A) 3-day old co-inoculated P. aeruginosa – E. coli biofilms (grid unit is 23.8 µm). B) Horizontal section near the base of the biofilm and vertical sections of the biofilm shown in panel A (scale bar = 20 µm). C) Distribution of P. aerugiosa and E. coli biomass as function of height, indicating that E. coli was the dominant species throughout a majority of the biofilm. E. coli appears red and P. aeruginosa-GFP appears green. Figure S6. Biofilm biomass on day 3 for the co-development of P. aeruginosa and E. coli biofilms subjected to different lo [file pone.0107186.s001.docx]

**Supplementary Information**


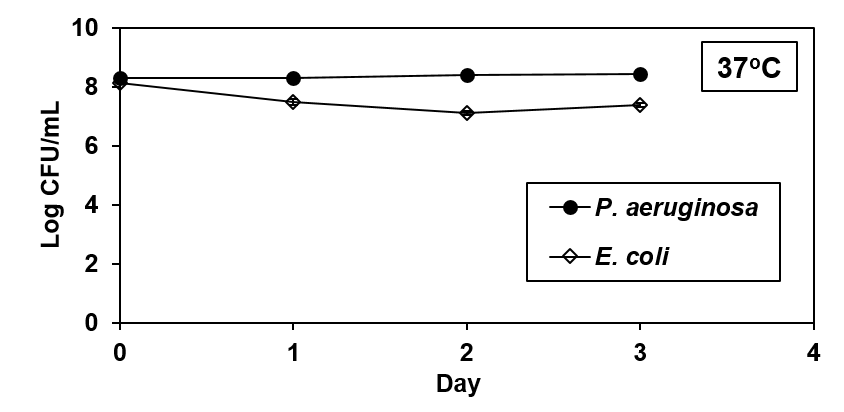


**Figure S1. Mean population density of *E. coli* and *P. aeruginosa* grown in R2A medium over a 3-day period at 37^o^C.** Both populations remained steady for the duration of the experiment, with *P. aeruginosa* exhibiting a higher population concentration.


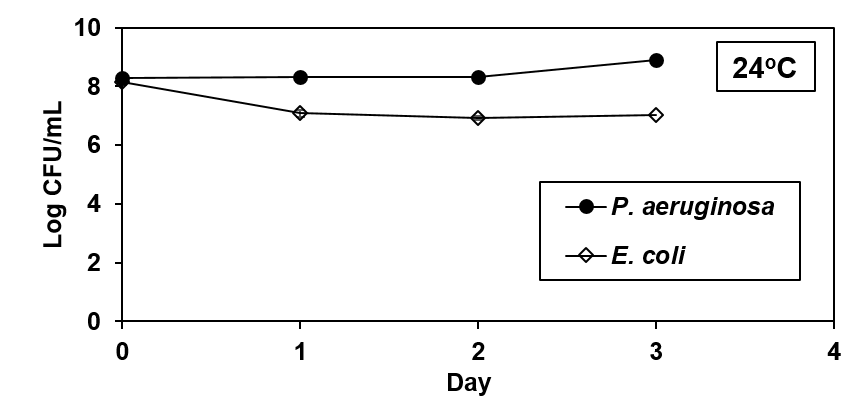


**Figure S2. Mean population density of *E. coli* and *P. aeruginosa* grown in R2A medium over a 3-day period at 24^o^C.** Both populations remained relatively steady for the duration of the experiment, with *P. aeruginosa* exhibiting a higher population concentration.


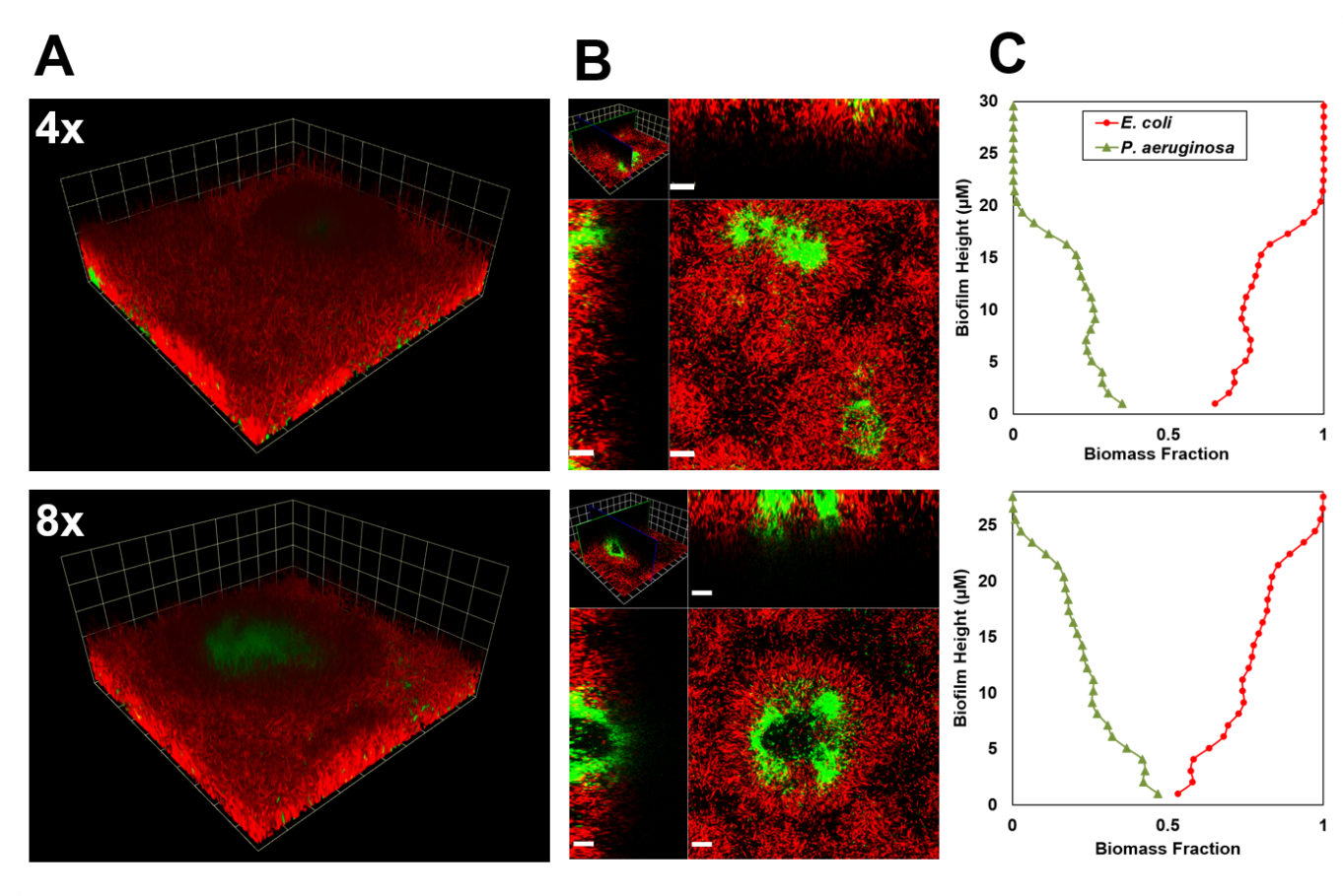


**Figure S3. Co-development of 3-day old *E. coli* and *P. aeruginosa* biofilms under varying R2A medium concentrations in a microfluidic flow cell.** Column: **A)** 3-day old co-inoculated *P. aeruginosa* – *E. coli* biofilms with under 4x and 8x R2A concentrations (grid unit is 23.8 μm). **B)** Horizontal section near the base of the biofilm and vertical sections of the biofilm shown in panel A (scale bar = 40 μm). **C)** Biomass fraction vs. biofilm height graph. Biofilms were counter-stained by SYTO 62. *E. coli* appears red and *P. aeruginosa*-GFP appears green.


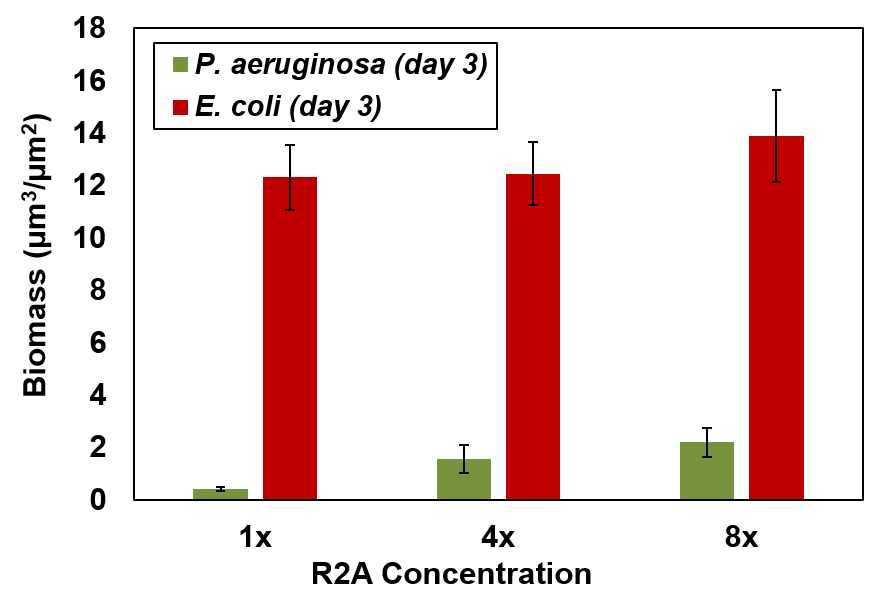


**Figure S4. Average biofilm biomass vs. R2A concentration for co-development of 3-day old *E. coli* and *P. aeruginosa* biofilms.**

**
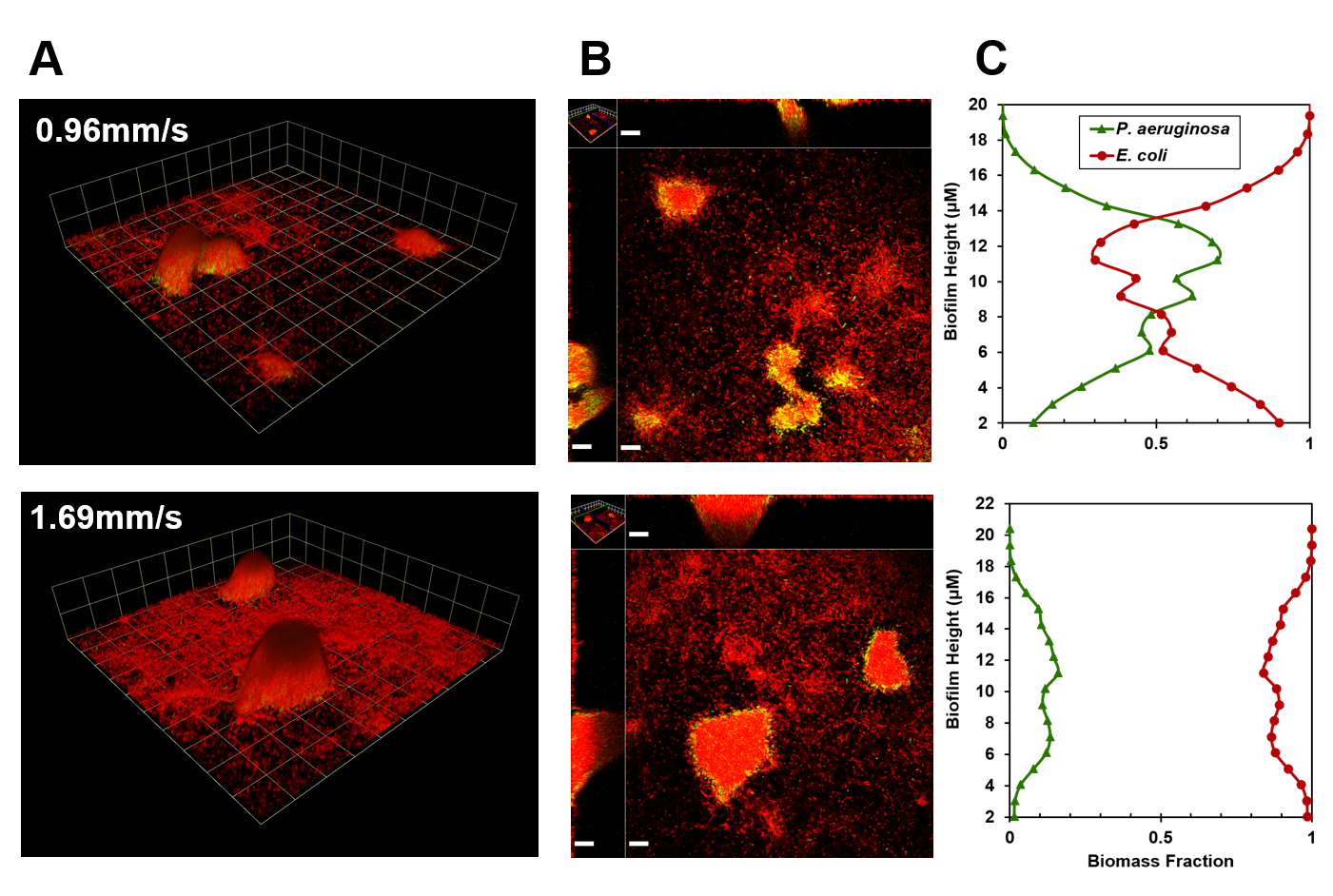
**

**Figure S5. Co-development of 3-day old *E. coli* and *P. aeruginosa* biofilms under a controlled flow gradient in a planar flow cell and in R2A medium.** Results are presented for two local fluid velocities, 0.96 mm/s (top row) and 1.69 mm/s (bottom row). Column: **A)** 3-day old co-inoculated *P. aeruginosa* – *E. coli* biofilms (grid unit is 23.8 μm). **B)** Horizontal section near the base of the biofilm and vertical sections of the biofilm shown in panel A (scale bar = 20μm). **C)** Distribution of *P. aerugiosa* and *E. coli* biomass as function of height, indicating that *E. coli* was the dominant species throughout a majority of the biofilm. *E. coli* appears red and *P. aeruginosa*-GFP appears green.


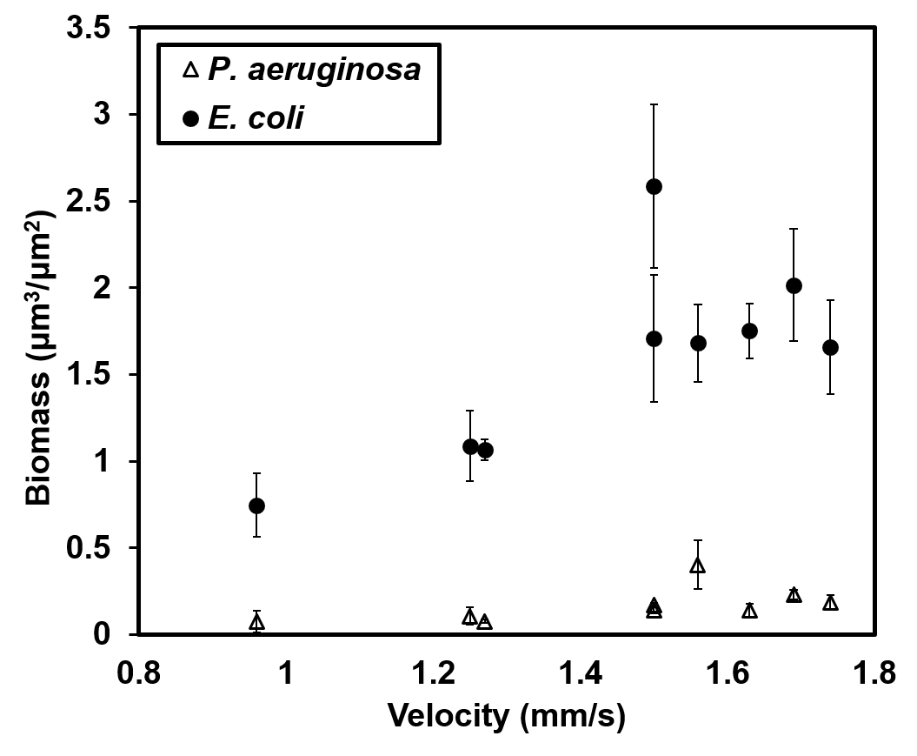


**Figure S6: Biofilm biomass on day 3 for the co-development of *P. aeruginosa* and *E. coli* biofilms subjected to different local velocities in R2A medium.**

**
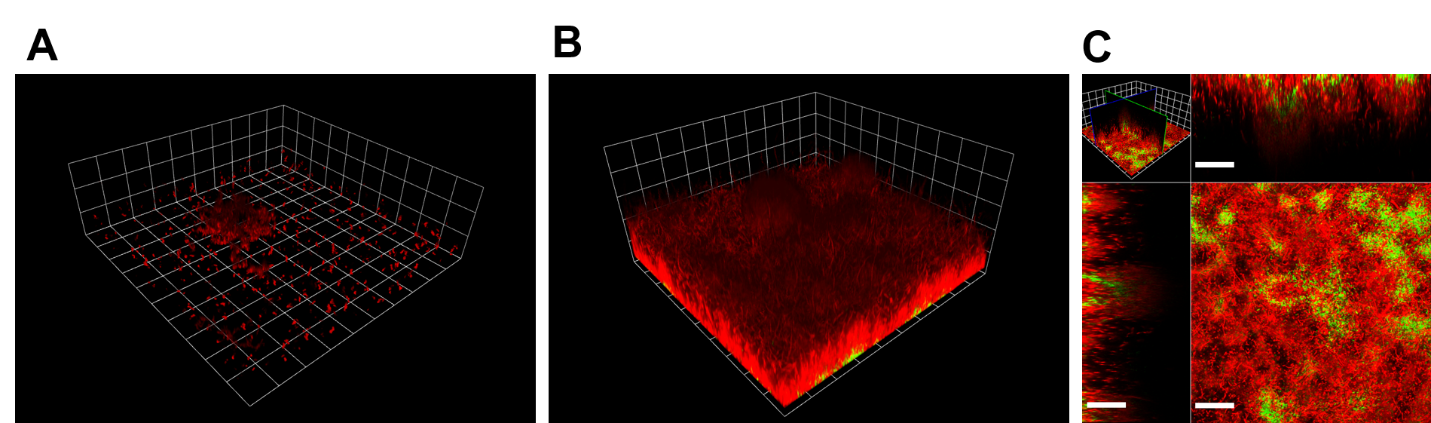
**

**Figure S7.** **Colonization of *E. coli* BW25113 biofilms by *P. aeruginosa* in R2A medium.** *E. coli* appears red and *P. aeruginosa*-GFP appears green or yellow. **A)** After 3-days of mono-species growth, *E. coli* formed sparse biofilms composed of small, isolated cell clusters (grid unit is 23.8 μm). **B)** Mixed *P. aeruginosa* and *E. coli* biofilm on day 6, after 3 days of mono-species *E. coli* growth plus 3 additional days of multi-species growth after inoculation of *P. aeruginosa*. (grid unit in B is 23.8 μm). Following introduction of *P. aeruginosa*, *E. coli* grew prolifically and adopted a configuration similar to that observed in other mixed-species experiments. **C)** Horizontal section near the base of the biofilm and vertical sections of the biofilm shown in panel B (scale bar = 40 μm).
